# Bioactive nanoparticle-based formulations increase survival area of perforator flaps in a rat model

**DOI:** 10.1371/journal.pone.0207802

**Published:** 2018-11-26

**Authors:** Ioana Lese, David Alexander Graf, Catherine Tsai, Adriano Taddeo, Martin Tobias Matter, Mihai A. Constantinescu, Inge Katrin Herrmann, Radu Olariu

**Affiliations:** 1 Department of Plastic and Hand Surgery, Inselspital, Bern University Hospital, Bern, Switzerland; 2 Department for Biomedical Research, University of Bern, Bern, Switzerland; 3 Faculty of Medicine, University of Bern, Bern, Switzerland; 4 Particles-Biology Interactions, Department of Materials Meet Life, Swiss Federal Laboratories for Materials Science and Technology (Empa), St. Gallen, Switzerland; BG Trauma Center Ludwigshafen, GERMANY

## Abstract

**Background:**

Distal flap necrosis is a frequent complication of perforator flaps. Advances in nanotechnology offer exciting new therapeutic approaches. Anti-inflammatory and neo-angiogenic properties of certain metal oxides within the nanoparticles, including bioglass and ceria, may promote flap survival. Here, we explore the ability of various nanoparticle formulations to increase flap survival in a rat model.

**Materials and methods:**

A 9 x 3 cm dorsal flap based on the posterior thigh perforator was raised in 32 Lewis rats. They were divided in 4 groups and treated with different nanoparticle suspensions: I–saline (control), II–Bioglass, III–Bioglass/ceria and IV–Zinc-doped strontium-substituted bioglass/ceria. On post-operative day 7, planimetry and laser Doppler analysis were performed to assess flap survival and various samples were collected to investigate angiogenesis, inflammation and toxicity.

**Results:**

All nanoparticle-treated groups showed a larger flap survival area as compared to the control group (69.9%), with groups IV (77,3%) and II (76%) achieving statistical significance. Blood flow measurements by laser Doppler analysis showed higher perfusion in the nanoparticle-treated flaps. Tissue analysis revealed higher number of blood vessels and increased VEGF expression in groups II and III. The cytokines CD31 and MCP-1 were decreased in groups II and IV.

**Conclusions:**

Bioglass-based nanoparticles exert local anti-inflammatory and neo-angiogenic effects on the distal part of a perforator flap, increasing therefore its survival. Substitutions in the bioglass matrix and trace metal doping allow for further tuning of regenerative activity. These results showcase the potential utility of these nanoparticles in the clinical setting.

## Introduction

Soft tissue reconstruction is a major part of plastic and reconstructive surgery. Based on the perforasome theory [1], perforator flaps can be raised almost in any region of the human body. Apart from the area supplied by the perforator, choke vessels between two perforasomes supply blood to the adjacent regions [2]. Tissue remodelling occurs through vasodilation and angiogenesis [3]. There is evidence suggesting that the sprouting of microvessels from a pre-existing network is triggered by an inflammatory environment [4]. Even though some of the principles involved in flap survival have been elucidated, distal flap necrosis remains one of the most feared complications in the clinical setting [5].

Several studies have investigated the use of exogenous agents, such as vascular growth factors and antioxidant small molecule drugs, in order to improve flap survival by inhibiting inflammation, decreasing oxidative stress and promoting angiogenesis [6–12]. While improvements have been reported, the application of growth factors such as vascular endothelial growth factor (VEGF) [13], basic fibroblast growth factor [6] and transforming growth factor beta [12] are restricted by their cost, short half-lives, and short-term effects. While costs can potentially be reduced by using antioxidant drugs to improve flap survival via VEGF-regulated pathways [7, 8, 14, 15], the treatment effect is still short-lived, and repeated dosing is required.

Nanotechnology offers exciting new therapeutic approaches, taking advantage of the size, structure and surface chemistry of nanoparticles, with their supposedly increased cellular uptake and ability to cross barriers that would be unreachable for other substance [16]. Interesting from a surgical point of view is that the mere augmentation of contact area by using nanoscale materials leads to strong adhesive forces. One manifestation of this phenomenon has recently been reported by Rose *et al*. [17] who spread metal oxide nanoparticles between two tissue pieces in order to glue them together (*nanobridging*). While these initial studies used silica and iron oxide nanoparticles, it has most recently been shown that bioglass-based nanoparticles exhibit exceptional tissue adhesive properties, thus opening the possibility to unify bioactivity with tissue adhesion.

Bioglass is a silica-based amorphous glass which has major applications in the field of bone tissue engineering [18, 19] and has more recently also been applied to soft tissue regeneration [20]. Bioglass-based polymer composites have been investigated for angiogenic properties *in vitro* and *in vivo* [21, 22]. The doping of the bioglass matrix with trace elements offers the possibility to further tweak the characteristics of the glass [23]. Many essential trace elements, such as strontium (Sr) and zinc (Zn), are well known for their anabolic effects [24, 25]. Strontium [26, 27] and zinc [28, 29] have both been shown to have angiogenic properties. Moreover, zinc has also been proven to exhibit anti-inflammatory effects [30, 31]. Silica-based nanoparticles, including bioglass, exhibit cytolytic activity at high concentrations. To reduce these effects, the use of hybrid nanoparticles containing bioglass and ceria has most recently been proposed based on *in vitro* cytotoxicity investigations [32]. Ceria has been reported to induce endothelial cell proliferation *in vitro* and vascular sprouting *in vivo* through VEGF induction [33, 34]. In addition to its angiogenic properties [33], ceria also acts as a reactive oxygen species scavenger, thereby reducing inflammation [32, 35]. By creating nanoparticle hybrids, the exceptional properties of bioglass can be unified with the cytoprotective and neoangiogenic properties of ceria. Furthermore, the therapeutic effects of zinc and strontium can be harnessed by matrix substitution and trace metal doping.

Here, we investigate the potential of bioactive bioglass-based nanoparticle formulations to increase flap survival in a rat model.

## Materials and methods

Bioglass, bioglass/ceria and zinc-doped strontium-substituted bioglass/ceria hybrid nanoparticles were produced by flame spray pyrolysis according to previously described methods [32]. Briefly, metal precursors were dissolved in 2-ethylhexanoic acid and diluted in acetonitrile or tetrahydrofuran to obtain a final concentration of 0.3 mol/L. The precursors were then fed into a spray nozzle at a flow rate of 5 mL/min, and dispersed by 5 L/min O_2_ and ignited by a CH_4_/O_2_ premix. Nanoparticles were then collected on a glass fibre filter mounted above the flame. As prepared, nanoparticles were characterized by X-ray diffraction, transmission electron microscopy and Inductively Coupled Plasma Optical Emission Spectroscopy. Nanoparticle suspensions were prepared by ultra-sonication immediately before application.

Thirty-two Lewis rats weighing 200–250 g were used for this study, which was approved by the Ethics Committee for Animal Experimentation, Bern, Switzerland (Approval number 89/16). All the animals were treated according to the Public Health Service Policy in Humane Care and Use of Laboratory Animals during the entire experiment.

### Surgical technique

All the operations were performed under continuous inhalation anaesthesia. Isoflurane 5% with oxygen (1 L/min) was used for the induction of anaesthesia (2–3 min) in an induction chamber. The rats were then placed in maintenance anaesthesia at 1–1.5% Isoflurane with 0.6 L/min oxygen. All the rats were maintained at a normal body temperature using thermal pads and treated with ophthalmic ointment to both eyes to prevent desiccation. All the groups received pre-emptive analgesia with Buprenorphine (50 μg/kg) subcutaneously, 30 minutes preoperatively.

**Fig 1:**
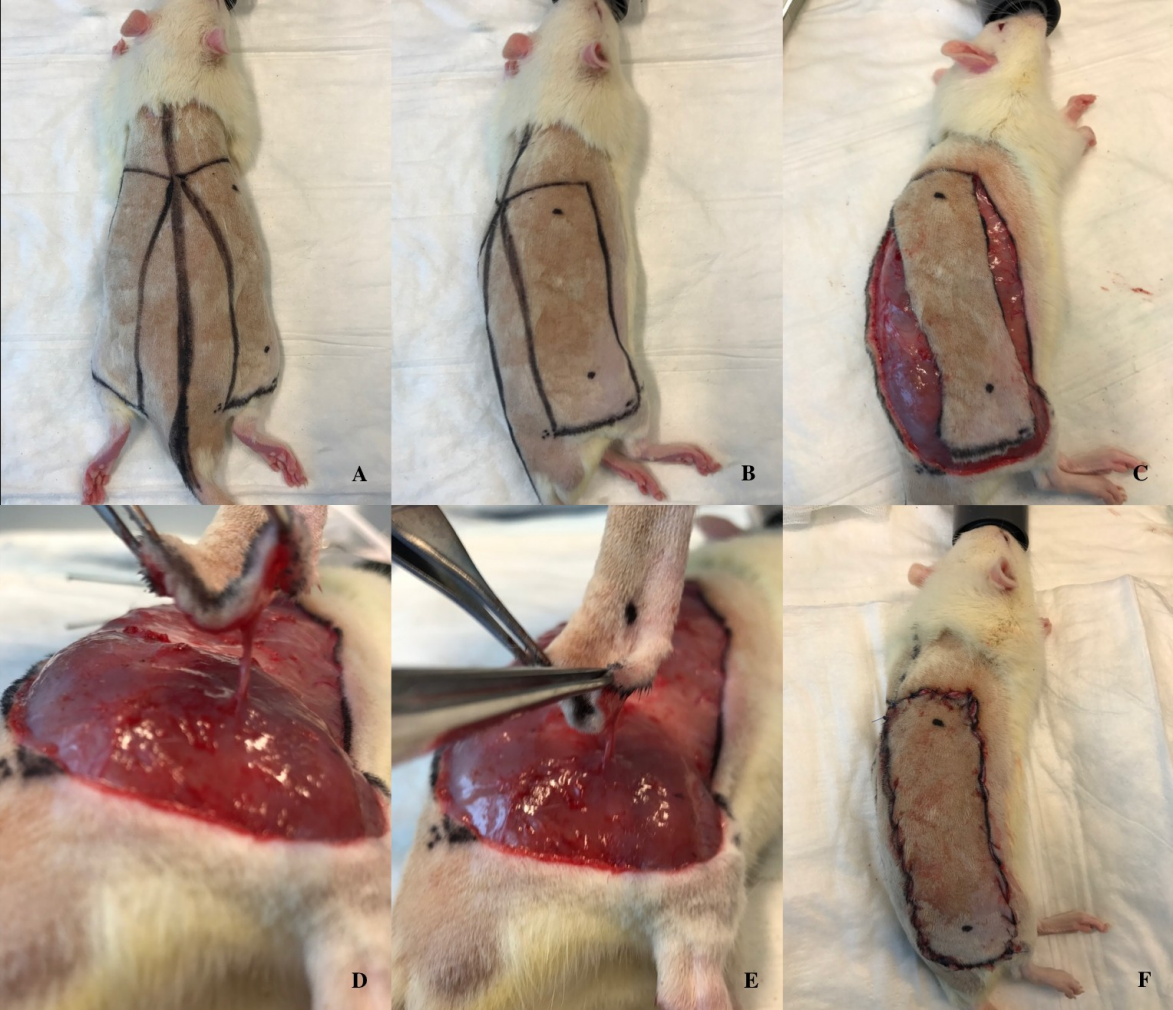
Operative technique. (A) Dorsal view of preoperative markings. (B) Lateral view of preoperative markings. (C) The flap was incised and raised at the subpannicular level. (D,E) Identification of the perforator arising through biceps femoris muscle. (F) Flap resutured in its original position.

**Fig 2:**
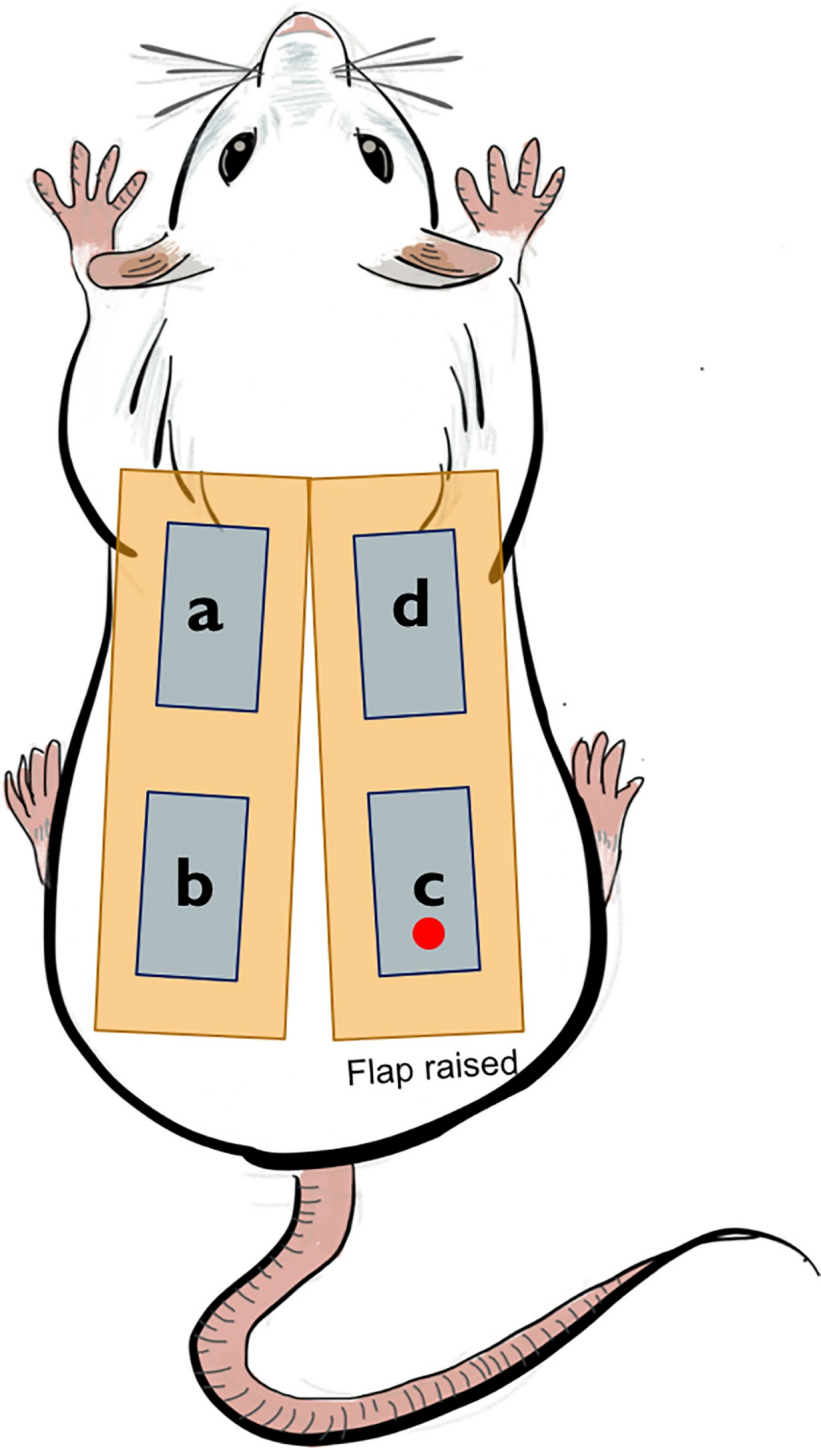
Schematic drawing of flap design. The right flap was incised and raised, while the left rectangle was used to collect healthy skin from the contralateral side. The red dot indicates approximate location of perforator. The four areas used for tissue harvesting are represented. Area *a* tissue sample was harvested on POD 0 and area *b*, *c*, *d* tissue samples were harvested on POD 7.

Based on the model described by Coskunfirat et al[36], we raised a posterior thigh perforator flap but without dissecting the musculocutaneous perforator up to its emergence from the femoral vessels, as depicted in Fig 1. The rats had their entire dorsum shaved and operative markings were drawn for all the animals on both halves: the dorsal midline was marked; then, a rectangle measuring 9 cm in length and 3 cm in width was designed bilaterally, beginning at a caudal line uniting the knee joint and the ischial tuberosity. Within the right rectangle, a point 2 cm distal from the caudal border of the flap, corresponding to the perforator location, and 1 cm proximal from the cranial border of the flap were marked on the middle axis of the flap. The right flap was incised and a retrograde flap elevation at the subpannicular level was performed until we identified the posterior thigh perforator, arising through the biceps femoris muscle. Conversely, the left side was not raised but used to sample healthy skin (region *a*) on the operation day, as well as region *b* on postoperative day (POD) 7 as envisioned in Fig 2. Within each of these rectangles, two smaller rectangular shapes, measuring 2x1 cm, were marked 1 cm apart from the proximal and distal border of the flap, respectively. After harvesting the *a* sample, the skin was closed with a simple running suture of Prolene 4–0. A second dose of Buprenorphine (50 μg/kg) was injected subcutaneously when starting to close the skin. Afterwards, the animals were allowed to fully wake up before returning them in their cages. Analgesia (Buprenorphine 50 μg/kg s.c.) was administered every 12 hours until the 2nd postoperative day. After that, the animals did not show any sign of pain and therefore didn`t need any further analgesia. The rats were observed for 7 days postoperatively and daily assessments of the health status were performed based on specifically developed score-sheet. All rats were euthanized on POD 7 by injecting 150 μg/kg Pentobarbital intraperitoneally. Skin samples *b*, *c* and *d* were harvested on POD 7.

### Nanoparticle treatment

The rats were randomly assigned to 4 experimental groups based on the type of treatment applied to the flap (Table 1).

**Table 1:** Group description.

Group Number	Group Acronym	Surgical operation	Treatment
I.	C	*Right side*: perforator flap *Left Side*: Healthy skin collection	Saline solution (Untreated control)
II.	Bg		Bioglass only nanoparticle solution
III.	Bg/Ce		Bioglass/ceria nanoparticle solution
IV.	Zn-Sr-Bg/Ce		Bioglass/ceria Zinc-doped strontium-substituted nanoparticle solution

In the control group, the flaps were sutured back on after applying 1 ml of saline. The treatment groups received 1 ml of suspension containing the corresponding types of nanoparticles (2.7 mg/ml). The nanoparticle dispersion volume of 1 mL was chosen because it fits well into the flap area of 3 x 9 cm^2^. The final concentration was adjusted to 0.1 mg of nanoparticles per cm^2^ flap area based on previous cytotoxicity evaluations[32]. The suspension was applied over the muscle fascia, right under the panniculum carnosum. The flaps were sutured back, first by fixing the corners with simple interrupted suture Prolene 4–0 and then with simple running suture Prolene 4–0.

### Digital planimetry

At the end of the experiment, on POD 7, the flaps were photographed from a distance of 40 cm with a Nikon D3300 camera with a Sigma 18–250 mm lens at 75mm focal length. The images were then processed with Adobe Photoshop®, which converted the images to bitmap and calculated pixel counts of viable flap area and of the total flap area. The viable area was selected as the part of the flap were the tissue colour was the same as the area overlapping the perforator. Moreover, the transition zone between viable and non-viable area was not included. The flap viability area was then calculated using the following formula:

$$Flap\;viability\;area=\frac{viable\;area\;(number\;of\;pixels)}{total\;area\;(number\;of\;pixels)}\times 100$$

### Laser–doppler flowmetry

Scanning Laser Doppler flowmetry with the Aïmago EasyLDI® Perfusion camera (Aïmago SA, Lausanne) was performed on POD 7 on the proximal and distal part of the flaps, focused on a point 2 cm distal from the caudal border of the flap, corresponding to the perforator location, and 1 cm proximal from the cranial border of the flap, on the middle axis, respectively. The results were expressed as mean perfusion units, with the distal point being expressed as percentage from the proximal point, taken as reference point.

### Histological evaluation

Tissue samples harvested from the *a* region (POD 0) and *d* region (POD 7) were analysed. At least 7 independent samples per experimental group were analysed in a blinded setting. Skin orientation (and direction of cutting) was kept the same for all samples. Sections were stained with hematoxylin-eosin. The epidermis, subcutis and muscle tissues were scored. Leucocyte infiltration, necrosis and other characteristics were assessed by a blinded certified pathologist and expressed as score with a range of 0–3. Endothelial activation was the only variable graded either 0 or 1. A necrosis score was obtained by adding the results obtained for acanthosis, epidermal necrosis, necrosis of the subcutaneous tissue and necrosis of the muscle, while the inflammation score resulted from adding leucocyte infiltration in epidermis, subcutaneous tissue and muscle.

### Systemic effect of nanoparticle treatment

Creatinine, alanine aminotransferase (ALAT) and aspartate aminotransferase (ASAT) were analysed from plasma collected on POD 7.

### Cytokine measurements

Protein extraction was performed as described in Duisit et al[37]. Briefly, skin samples were weighed on an analytical scale and cut into small pieces and transferred into M tubes (Miltenyi Biotec GmbH, Bergisch Gladbach, Germany) containing RIPA buffer (50mM Tris-HCl, pH 8.0, with 150mM sodium chloride, 1.0% Igepal CA-630 (NP40), 0.5% sodium deoxy cholate and 0.1% sodium dodecyl sulfate) and protease inhibitor cocktail (Sigma). M tubes were put on a gentle MACS Dissociator (Miltenyi Biotec GmbH) and homogenized using the program specific for protein extraction. Homogenates were incubated on ice, underwent sonication and, after a short spin down, the supernatants were transferred to smaller tubes for centrifugation at 13’000 rpm for 1 hour at 4°C. Again, the supernatant was transferred to a new tube for further analysis. For protein quantification, a classical Bicinchoninic acid (BCA) assay was performed. A bovine serum albumin standard (2.0mg/ml in 0.9% aqueous NaCl containing sodium azide, Thermo Fisher Scientific) was prepared and transferred to a flat transparent 96-well plate (Thermo Fisher Scientific). 5 μl of sample were added to the wells and the assay was performed according to the DC Protein Assay kit instructions (Bio-Rad, Hercules, CA, USA). The OD was measured at 750nm on a plate reader (Infinite M1000 PRO) and interpolated by GraphPad Prism 7 software. Luminex multiple assay (ProcartaPlex Mix&Match Rat 7-Plex) was performed on tissue samples (as shown in Fig 1) and plasma collected on POD 0 and 7 for the following proteins: vascular endothelial growth factor A (VEGF-A), soluble vascular cell adhesion protein 1 (vCAM-1), interleukin-1β (IL-1β), interleukin-6 (IL-6), intercellular adhesion molecule 1 (iCAM-1) and monocyte chemoattractant protein-1 (MCP-1). The procedure was performed based on the original manufacturer instruction manual as described by Müller et al[38] and plates were read on a FLEXMAP 3D system (Luminex, Austin, CA, USA).

Immunofluorescence stainings were performed for CD31 and vascular endothelial (VE)-Cadherin on tissue samples taken on POD 0 (*a)* and POD 7 (*b*, *c* and *d*), following the protocol described by Zhang et al [39]. Skin samples from the different regions were rinsed in PBS, blotted dry, embedded in OCT matrix (Tissue-Tek; Sakura Finetek Europe BV, Leiden, The Netherlands), and stored at −25°C until cryosections (5 μm tick) were made. Sections were fixed with aceton and stained with biotin anti-rat CD31 (Miltenyi Biotec) or goat anti-rat-VE-Cadherin (Santa Cruz Biotechnology, Dallas, US) and Streptavidin Cy3 (Sigma-Aldrich) or Donkey anti-goat IgG—Alexa Fluor 488 (Molecular Probes, Oregon, US) as secondary reagent. Images were taken with a fluorescent microscope (Leica DMI 4000B; Leica Microsystems Schweiz AG, Heerbrugg, Switzerland) and analyzed by ImageJ (National Institutes of Health, Bethesda, Md) and GraphPad Prism 7 software. Integrated fluorescence densities were quantified in three to five images for each sample, the mean of which was used for statistical comparison between groups.

### Statistical analysis

The Graph Pad Prism 7 software (Graph Pad Software Inc., La Jolla, CA, USA) was used for statistical analyses and all values were marked as mean ± standard error. For normally distributed data, multiple comparisons within the same group were performed using repeated measures one-way ANOVA and Tukey’s multiple comparison test for correction. Comparisons between different groups were performed with the one-way ANOVA, using Dunnett’s multiple comparison test for correction. For the non-normally distributed data, the Kruskal-Wallis test, together with Dunn`s test for correction were employed. Fisher`s exact test was used for categorical variables. P values < 0.05 were considered to indicate statistical significance.

## Results

### Influence of nanoparticle treatment on flap survival

**Fig 3:**
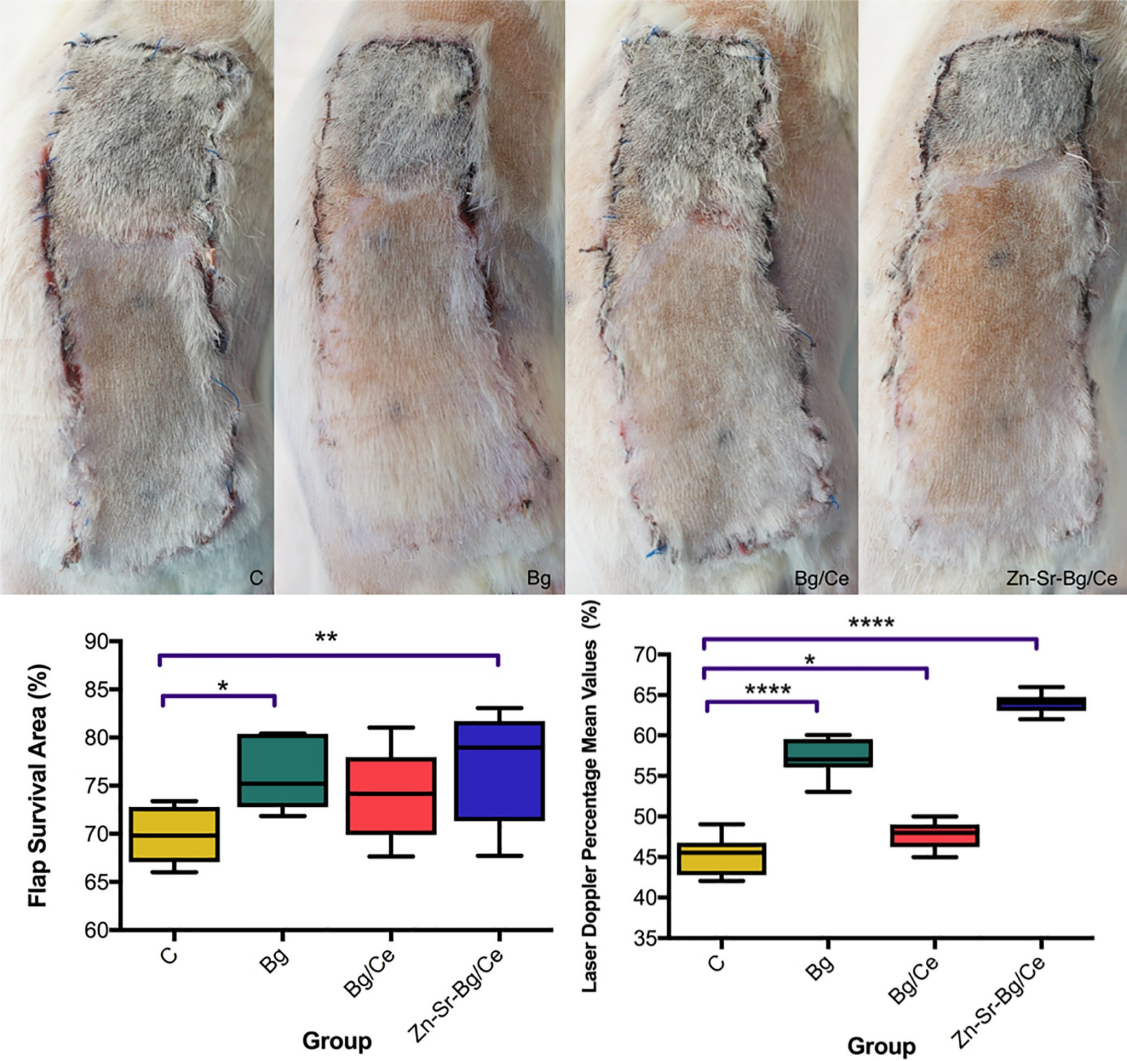
Nanoparticle treatment promotes flap survival. (A) Representative pictures showing the macroscopic appearance of the flaps in the 4 groups on POD7. (B) Flap survival analysed by digital planimetry (left graph) and Laser-Doppler flowmetry (right graph) in the four treatment groups on POD 7. Data are presented as mean, with the whiskers extending to the minimum and maximum values. * p<0.05, ** p < 0.01, *** p< 0.001, **** p<0.0001.

Flap survival was evaluated at POD 7 and compared to control. All flaps showed distal necrosis (Fig 3A). Digital planimetry was performed to calculate the percentage of viable tissue (Fig 3B). Untreated flaps (Group C) showed a 69% average survival rate. A significant increase in flap survival was observed after treatment with Bg and Zn-Sr-Bg/Ce nanoparticles (76 and 77.3% average survival, respectively). Increased survival was observed also in Bg/Ce treated flaps (74% average survival), but did not reach statistical significance.

Evaluation of blood flow by laser Doppler performed at POD 7 showed that the distribution of the blood flow followed the same trend as flap survival (Fig 3B). The control group showed a mean flow of 45.3% from the reference point. The Bg/Ce group (47.8%), the Bg group (57.1%) and the Zn-Sr-Bg/Ce group (63.9%) showed a statistically significant higher flow when compared to the C group.

### Histological analysis of skin flaps

**Fig 4:**
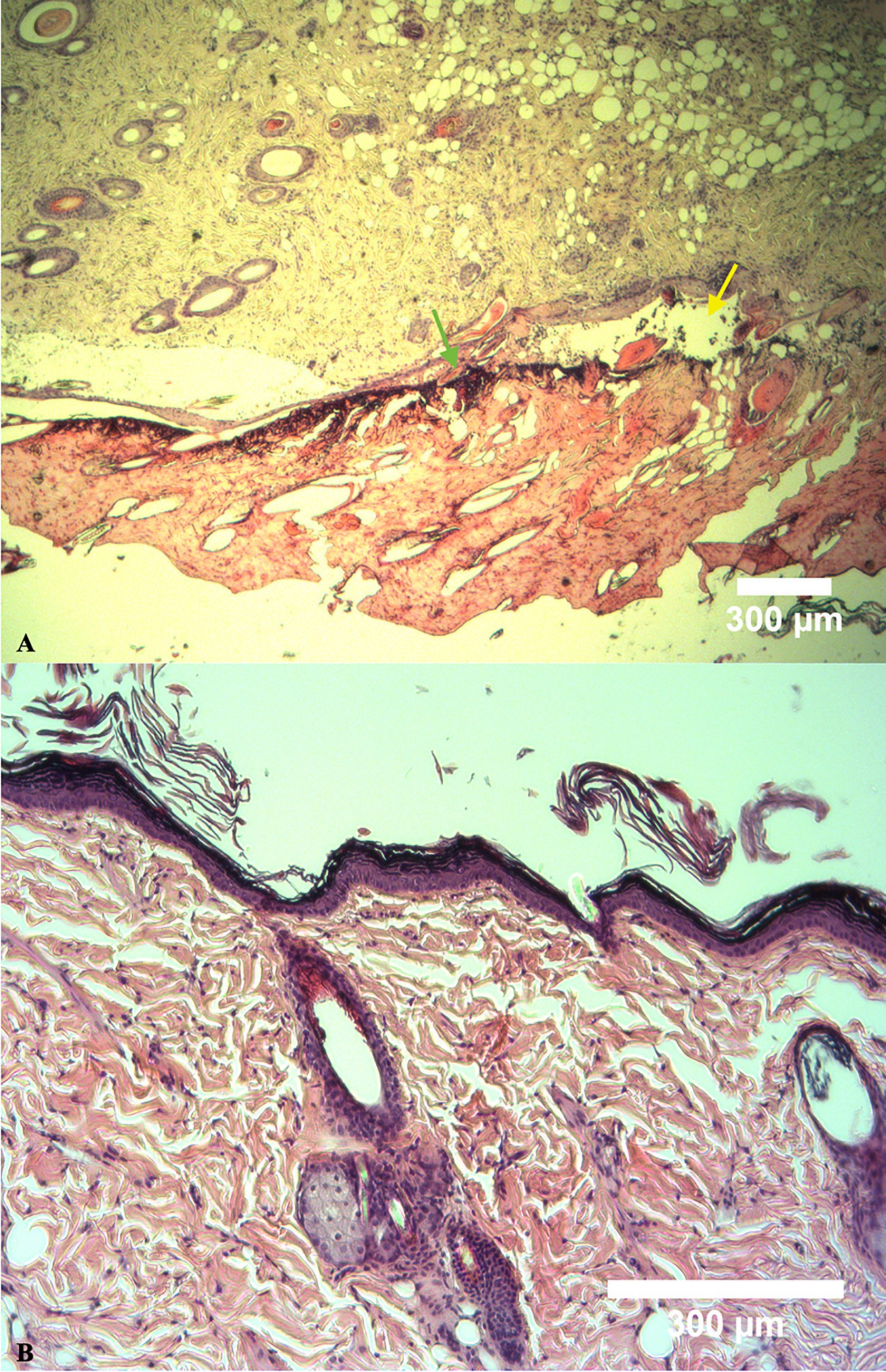
Histological differences between control group and the group treated with Zn-Sr-Bg/Ce nanoparticles. (A) Representative histological picture of region *d* from C group (saline-treated): Hyperkeratosis at the bottom of the image, with epidermal necrosis (green arrow) and pustule with inflammatory infiltrate (yellow arrow). Hair follicles are only present in the left corner, while the right corner shows follicle atrophy. (B) Representative picture of region *d* from Zn-Sr-Bg/Ce treated group: epidermal and subcutaneous tissue with hair follicles, sebaceous glands and collagen fibers.

The results are presented in Table 2. The necrosis score was significantly lower for the Zn-Sr-Bg/Ce group and a trend towards attenuated necrosis was also found in the other two treatment groups, as compared to control group. Regarding the inflammation score, we observed a reduced leucocyte infiltration in all the groups when compared to the C group, but statistical significance was reached only in the Zn-Sr-Bg/Ce group. Fig 4 illustrates histological differences between group C and Zn-Sr-Bg/Ce.

**Table 2:** Histological analyses of the distal part of the flaps (region *d)*.

Localisation	Characteristic	C	Bg	Bg/Ce	Zn-Sr-Bg/Ce
***Epidermis/dermis***	Pustels	1.75 (±0.37)	0.4286 (±0.3) *	0.25 (±0.25) *	0.875 (±0.35)
	Acanthosis	2 (±0.33)	0.29 (±0.29) **	1.12 (±0.3)	1.25 (0.25)
	Epidermal hypertrophy	0.5 (±0.33)	0.28 (±0.28)	0.57 (±0.2)	0.87 (±0.22)
	Epidermal necrosis	2.37 (± 0.42)	1.57 (±0.37)	1.75 (±0.45)	1 (±0.38)
	Leucocyte infiltration	1.62 (±0.26)	1 (±0.22)	1.12 (±0.3)	1 (±0.27)
	Follicle atrophy	1 (±0.5)	0.14 (±0.14)	1.71 (±0.47)	1 (±0.32)
	Vessel/Endothelial cells	0.75 (±0.25)	1.57 (±0.2)	1.87 (±0.12) **	1 (±0.19)
***Subcutaneous tissue***	Necrosis	1.5 (±0.38)	1.71 (±0.36)	1.5 (±0.42)	0.62 (±0.42)
	Leucocyte infiltration	1.875 (±0.23)	1.43 (±0.3)	1.37 (±0.26)	1.12 (±0.3)
	Granulation tissue	2.62 (±0.18)	2.14 (±0.4)	2.25 (±0.25)	1.62 (±0.18) *
***Muscle***	Necrosis/degeneration	1.75 (±0.31)	2 (± 0.22)	1.85 (±0.14)	0.75 (±0.41)
	Atrophy	1.86 (±0.14)	0.28 (±0.28) **	0.28 (±0.18) **	0.12 (±0.12) ***
	Leucocyte infiltration	1.85 (±0.26)	2 (±0.3)	2.28 (±0.18)	0.87 (±0.4)
	Endothelial activation	0.13 (±0.12)	0.57 (±0.2)	0.75 (±0.16)*	0.75 (±0.16)*
	Necrosis score	7.62 (±1.5)	5.57 (±0.72)	6 (±0.94)	3.62 (±1.2) *
	Inflammation score	6.87 (±0.87)	4.85 (±0.4)	4.75 (±0.77)	3.87 (±0.9) *

Values are expressed as mean ± standard error of the mean.

* p<0.05

** p<0.01

*** p<0.001

ns–not significant.

### Systemic effect of nanoparticle treatment

**Fig 5:**
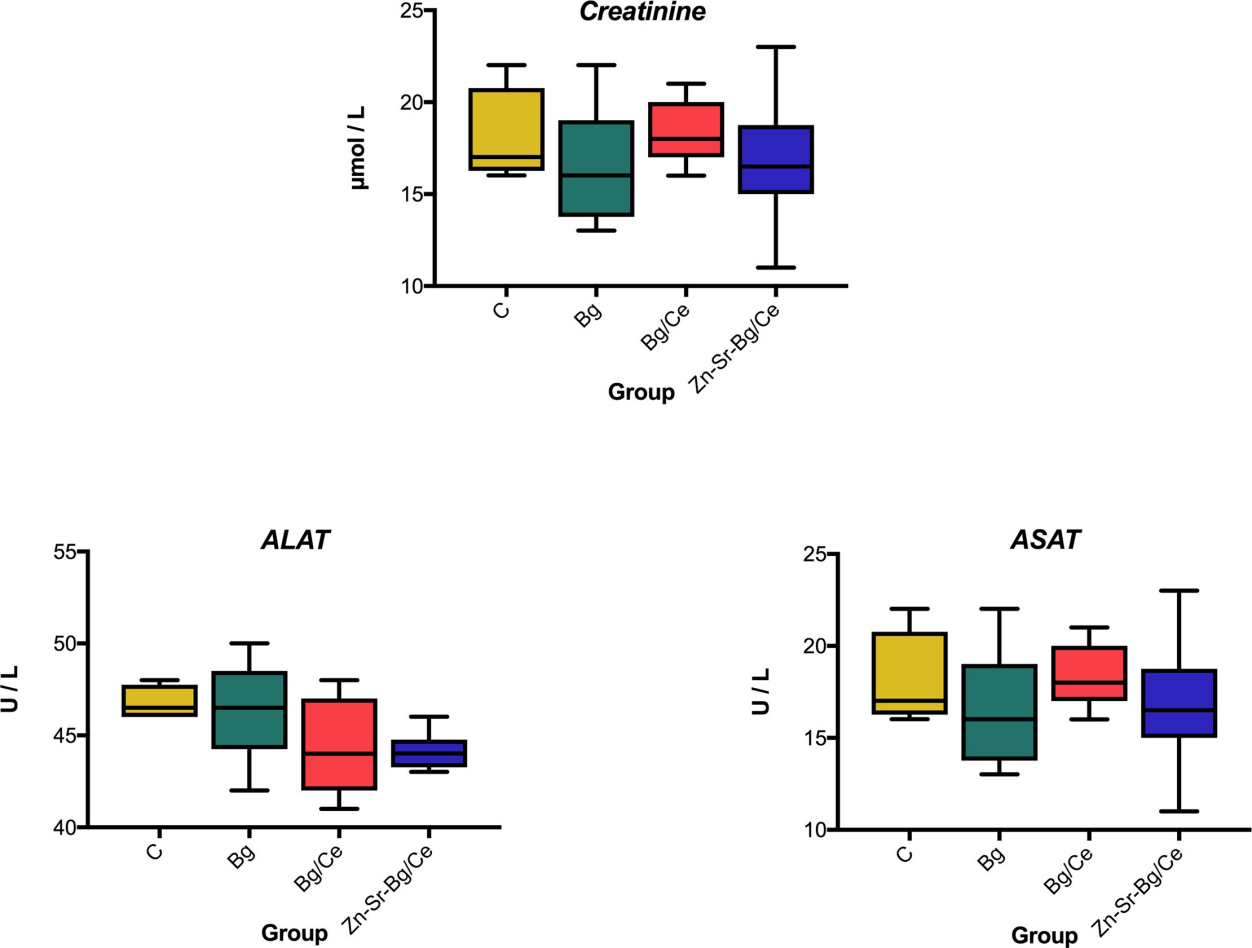
Nanoparticle treatment does not induce systemic toxicity. **Biochemical analyses of the plasma levels of creatinine, alanine aminotransferase (ALAT) and aspartate aminotransferase (ASAT) collected at POD7 from the four treatment groups.** Data are presented as mean, with the whiskers extending to the minimum and maximum values.

Nanoparticle treatments did not induce any statistically significant differences in the plasma levels of creatinine, ALAT and ASAT, as shown in Fig 5.

### Biochemical analysis of skin flaps and plasma

Laboratory analyses are presented as normalized ratio calculated by dividing the values obtained from region *b*, *c*, and *d* of all groups by the mean of combined raw *a* values from all groups.

**Fig 6:**
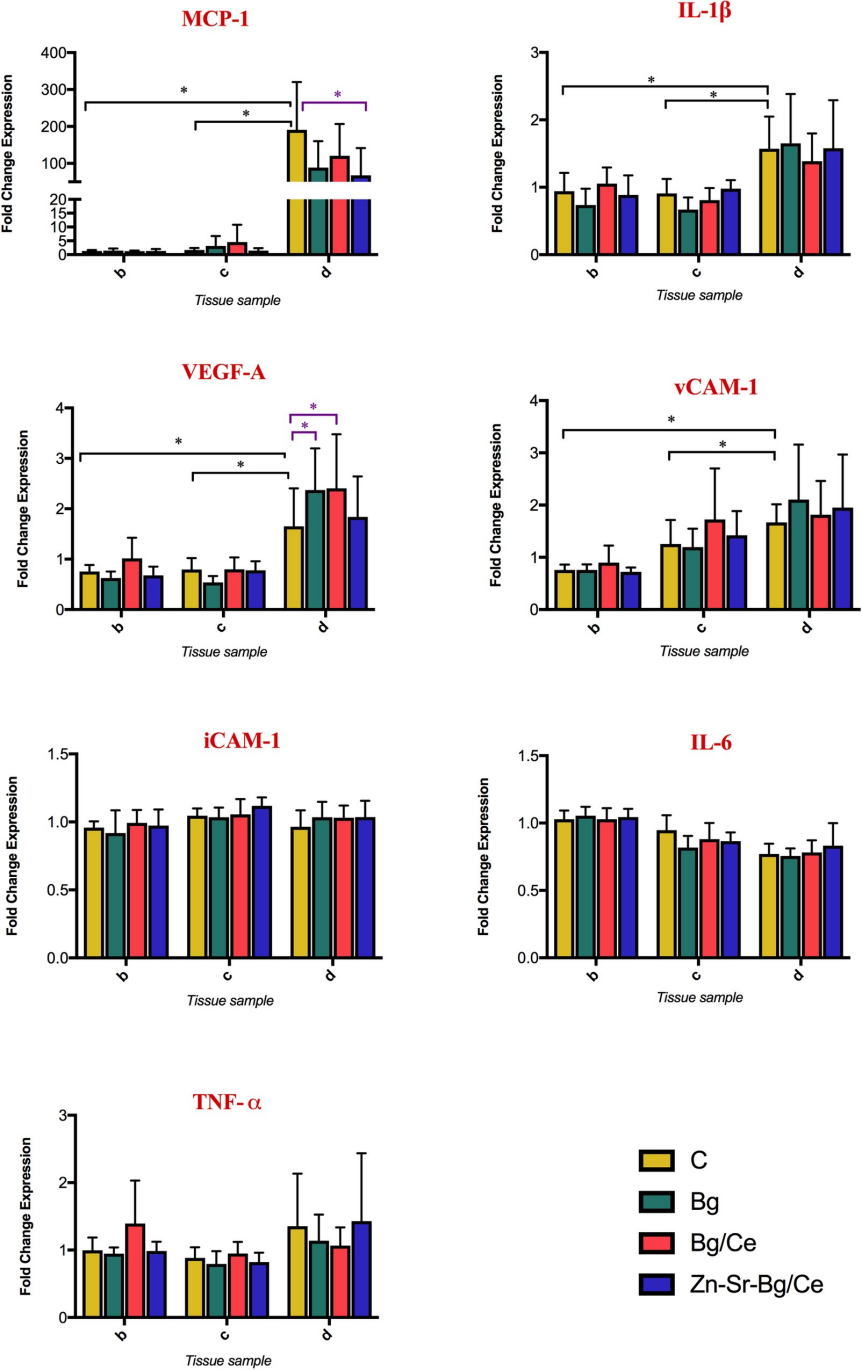
Effects of nanoparticle treatment on tissue expression of inflammatory and endothelial cell relate cytokines. Cytokine levels in the skin collected on POD7 from the different flap areas (see Fig 1) measured using a Luminex-like assay. Data are presented as fold change of cytokine expression in each region with respect to region *a*. Bars show mean and standard deviation. *p<0.05.

**Fig 7:**
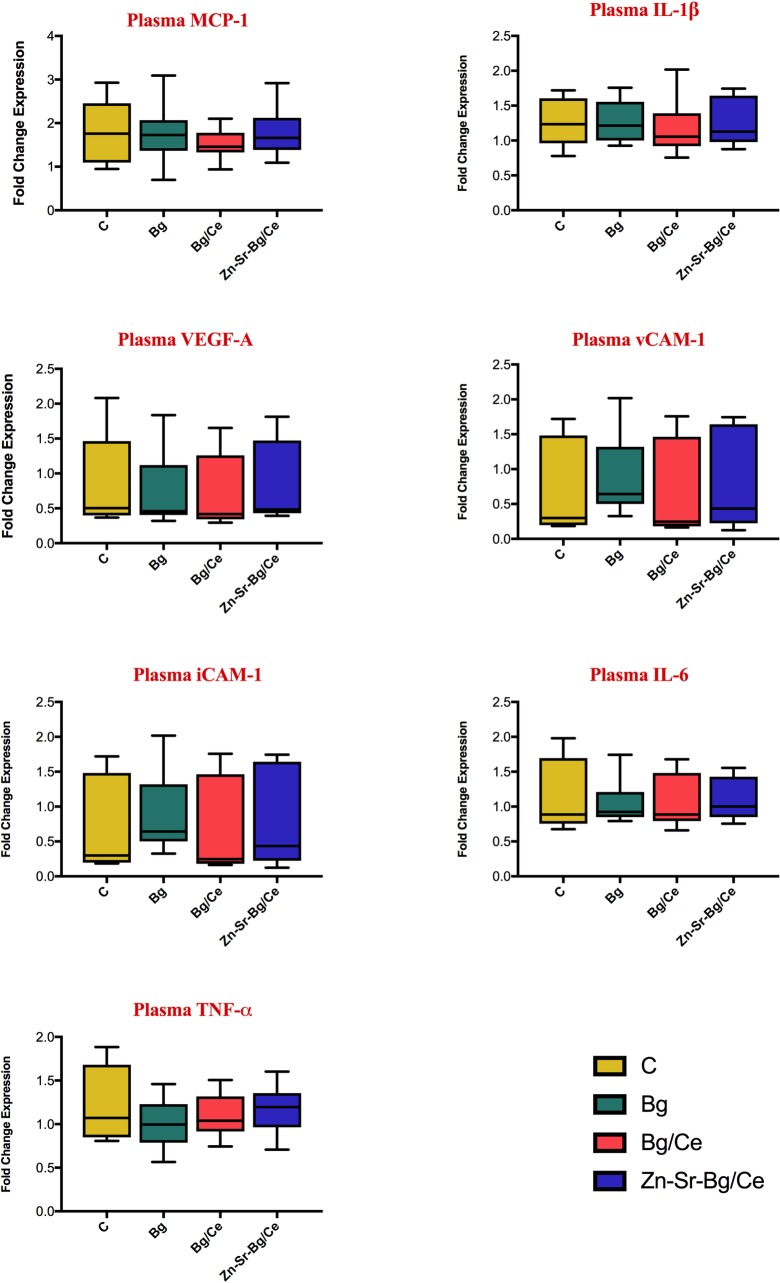
Effects on nanoparticle treatment on plasma expression of inflammatory and endothelial cell relate cytokines. Cytokine levels measure in plasma on POD 7 measured using a Luminexlike assay. Data are presented as fold change of cytokine expression in the plasma with respect to plasma collected at POD7. Bars show mean, with the whiskers extending to the minimum and maximum values.

The expression of IL-1ß, IL-6, MCP-1, TNF-α, VEGF-A, soluble VCAM-1 and ICAM-1 in skin sample was measured to evaluate the effect of the nanoparticles in regulating inflammation and endothelial cell response. As illustrated in Fig 6, a significant increase of MCP-1, IL-1β, VEGF-A and soluble VCAM-1 was observed in the control group in the distal necrotic skin of the flap (region *d*) as compared to healthy skin (region *b*) and non-necrotic flap skin (region *c*), close to the perforator. Expression of soluble iCAM-1, IL-6 and TNF-α remained unaltered in the examined areas. Nanoparticle treatments reduced the levels of MCP-1 in the necrotic skin (region *d*) as compared to untreated control, independently of the kind of nanoparticles used. Statistically significant difference was however reached only by the Zn-Sr-Bg/Ce group. Moreover, we observed a further significant increase of VEGF-A expression in region *d* in Bg and Bg/Ce treated rats. The levels of IL-1β and VCAM-1 were not changed by any of the nanoparticles used. Notably, there were no statistically significant differences between cytokine levels in blood plasma, as shown in Fig 7.

**Fig 8:**
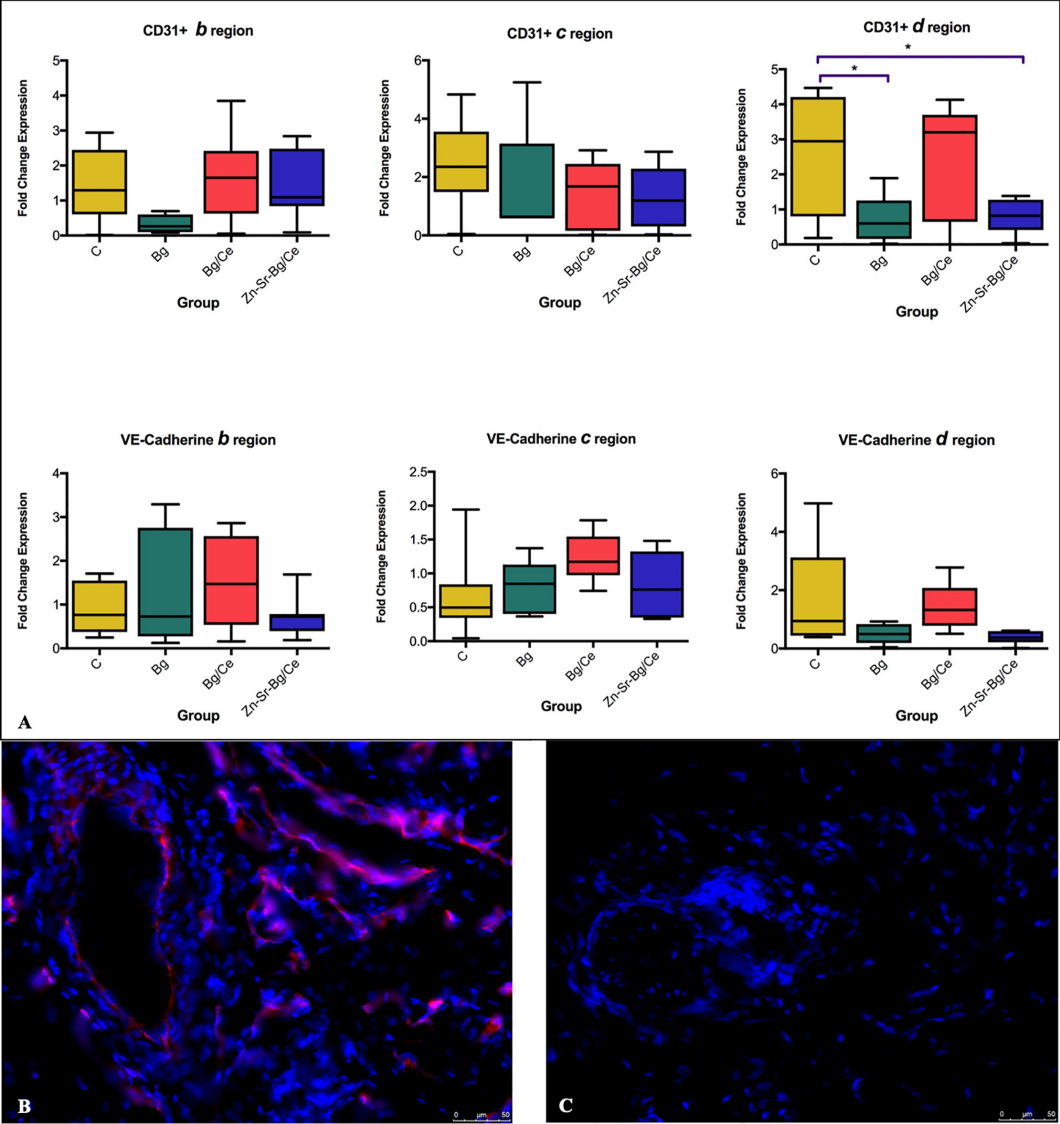
Effect of nanoparticle treatment on skin endothelial cells. (A) Quantification of tissue fluorescence intensity of CD31 and VE-Cadherin expression measured with Fiji Image J software Data are presented as fold change of raw integrated density of CD31 and VE-Cadherin expression in each region with respect to region *a*. Bars show mean, with the whiskers extending to the minimum and maximum values. Representative pictures showing the expression of CD31 in the region *d* in the control group (B) and the group treated with Zn-Sr-Bg/Ce nanoparticles (C). *p<0.05.

In order to better investigate endothelial cell response under nanoparticle treatment, CD31 and VE-Cadherin expression was evaluated by immunofluorescence staining of the skin. Expression of CD31 was increased in the distal necrotic part of the flap (region *d*) as compared to region *b* with respect to the control group. Treatment with Bg and Zn-Sr-Bg/Ce groups showed reduced expression of CD31 on POD7 in region *d*, while Bg/Ce did not show any significant difference. VE-Cadherin expression remained constant, also in the necrotic part of the flap and was not affected by any of the treatment regimens (Fig 8A). Fig 8B and 8C show the intensity of CD31 staining in region *d* of the C and Zn-Sr-Bg/Ce groups, respectively.

## Discussion

In the current study, we report on the promising beneficial effects of bioactive nanoparticle-based formulations on skin flap survival in a rat model. The current study is, to the best of our knowledge, the first report on the prospective use of metal oxide nanoparticle-based formulations to improve skin flap survival. Overall, the application of bioglass-based nanoparticle formulations significantly improved flap survival. With regard to the underlying mechanism, there are strong indications of an attenuated inflammatory response (decreased MCP-1and CD31 expression), as well as a neo-angiogenic effect (increased VEGF-A expression) exerted by the nanoparticles. These modulations have led to reduced necrosis and better flap survival due to improved perfusion, as confirmed by laser Doppler analyses.

Pertaining to the molecular pathways involved in flap survival, inflammatory cytokines play an important role. MCP-1, a key chemokine that regulates migration and infiltration of monocytes/macrophages, also plays an important role in the induction of new blood vessels during wound repair and inflammation [40]. MCP-1 expression in the necrotic part of the skin flap was strongly upregulated in the control group. In the nanoparticle treated groups with increased flap survival, MCP-1 levels were lower than the corresponding control group. Qing *et al*. reported increased levels of MCP-1 in the flap areas where growth of choke vessels was detected, thereby leading to increased vascularisation of the distal part of the flap [5]. In their study, peak values of MCP-1 were recorded on POD 5 with decreasing MCP-1 concentrations on POD 7, indicating a decline in MCP-1 expression once new vessels have formed, which might again be in line with our findings at POD 7. IL-1β, another pro-inflammatory cytokine, recorded constant levels in plasma and only marginally higher levels in region *d* compared to *b* and *c*. Wang *et al*. found that low levels of IL-1β in plasma and tissue samples were correlated with a better flap survival [9]. In our study, the nanoparticle-treated flaps consistently showed attenuated inflammation compared to control group, as indicated by the lower levels of MCP-1 and reduced leukocyte infiltration in the groups with better flap survival.

There is strong evidence that the most effective strategy in promoting flap survival is increasing blood supply. In our study, VEGF levels remained constant in region *b* and *c*, but a clear difference could be seen in the distal part of the flap (*d*), where groups Bg and Bg/Ce recorded higher levels of VEGF-A, therefore suggesting a stimulating effect of the nanoparticles on endothelial cells. A trend toward better angiogenesis through increased levels of VEGF-A was also found for the Zn-Sr-Bg/Ce group. While there are discrepancies between various studies in terms of mechanisms and cytokine regulation, the bioactive nanoparticles used in our study appear to partly increase flap survival via VEGF-A induction. This effect was also confirmed by histological analyses, which showed an increased score of vessel/endothelial cells, as well as an increased endothelial activation, with statistically significant differences seen in the Bg and Bg/Ce groups.

With regard to CD31 expression, Jafari *et al*.[10] showed a correlation between increased numbers of CD31+ vessels and improved flap survival. In our model, the necrotic area of the flap (region *d*) presented elevated expression of CD31 in untreated rats. This expression was significantly reduced in the Bg and Zn-Sr-Bg/Ce groups. These results, together with stable expression of VE-Cadherin in all the groups and areas, suggest that endothelial cell response is strongly induced in the necrotic area and that inflammation is partially reduced on POD7 in the Bg and Zn-Sr-Bg/Ce groups, in which flap survival was better. Although there were no differences in VE-Cadherin among the groups, this finding could actually suggest the lack of endothelial disruption by the nanoparticles, indicating good biocompatibility of these nanoparticles.

The main limitations of our study are: (1) the dynamic changes of the tissue sample cytokines could not be analysed in vivo, (2) we did not investigate the possibility of the long-term effect of the nanoparticles and (3) the exact mechanisms underlying the distinct effects of these nanoparticle-based formulations and their distribution have yet to be further assessed.

While the nanoparticle treatment led to improved flap survival, a feared disadvantage of nanoparticles is the limited knowledge regarding systemic effects [41]. The lack of differences in plasma cytokine levels, as well as in organ damage markers, such as creatinine, ALAT and ASAT, indicates that the nanoparticles act primarily locally, without any detectable systemic effect, as reported also by Reinert *et al* [42]. However, a thorough understanding of the exact mechanism of action and potential long-term (side) effects is of paramount importance for future clinical applications.

## Conclusions

This study is a promising first example of the potential of nanoparticle-based formulations in soft tissue survival in the field of plastic and reconstructive surgery. These novel formulations show local anti-inflammatory and neo-angiogenic properties translating to significantly increased distal flap survival, with no detectable adverse side effects. Therefore, the incorporation of such bioactive inorganic nanoparticle-based formulations into therapeutic management of perforator flaps would be a logical next step in the clinical research with major prospective implications in reconstructive surgery.

## Supporting information

S1 File Dataset. (XLSX)
